# Plant virus infections control stomatal development

**DOI:** 10.1038/srep34507

**Published:** 2016-09-30

**Authors:** Rose R. Murray, Mark S. M. Emblow, Alistair M. Hetherington, Gary D. Foster

**Affiliations:** 1 School of Biological Sciences, Life Sciences Building, University of Bristol, 24 Tyndall Avenue, Bristol BS8 1TQ, UK

## Abstract

Stomata are important regulators of carbon dioxide uptake and transpirational water loss. They also represent points of vulnerability as bacterial and fungal pathogens utilise this natural opening as an entry portal, and thus have an increasingly complex relationship. Unlike the situation with bacterial and fungal pathogens, we know very little about the role of stomata in viral infection. Here we report findings showing that viral infection influences stomatal development in two susceptible host systems (*Nicotiana tabacum* with TMV (*Tobacco mosaic virus*), and *Arabidopsis thaliana* with TVCV (*Turnip vein-clearing virus*)), but not in resistant host systems (*Nicotiana glutinosa* and *Chenopodium quinoa* with TMV). Virus infected plants had significantly lower stomatal indices in systemic leaves of susceptible systems; *N. tabacum* 9.8% reduction and *A. thaliana* 12.3% reduction, but not in the resistant hosts. Stomatal density in systemic leaves was also significantly reduced in virus infected *A. thaliana* by 19.6% but not in *N. tabacum* or the resistant systems. In addition, transpiration rate was significantly reduced in TMV infected *N. tabacum*.

Stomata are microscopic pores located on the aerial parts of terrestrial plants and consist of a central pore surrounded by two guard cells. When the guard cells are fully turgid the pore gapes open and this permits the uptake of CO_2_ for photosynthesis and the loss of water by evapotranspiration. This latter process is the driving force responsible for the transport of water and mineral nutrients from the roots to the aerial parts. When the guard cells lose turgor the pore closes and this has significance during, for example, periods of reduced soil water availability as it allows the plant some degree of water conservation. Stomatal aperture is controlled by an array of internal cues and environmental signals that serve to “set” stomatal apertures to suit the prevailing environmental conditions^1^,^2^,^3^. Stomatal development is also regulated by environmental signals and this plays out as changes in leaf stomatal density and index^4^,^5^,^6^. Light and atmospheric CO_2_ concentration exert systemic control of stomatal development with signals from old leaves contributing to the control of stomatal development in new developing leaves^7^,^8^,^9^. In addition to changes in stomatal aperture, alterations in stomatal density and index impact on plant water use efficiency^10^,^11^,^12^.

Stomata also allow pathogens, such as rust fungi, bacteria and nematodes, to gain access to the plant and in recent years it has been recognized that stomata form part of the plants’ innate immune system^13^,^14^,^15^. In contrast to the situation with fungi and bacteria, very little is known about whether viral infection influences stomatal aperture or development. Here we report the result of experiments showing that viral infection of susceptible hosts results in reductions in stomatal density, stomatal index and reduced transpirational water loss.

## Results

### Stomatal index and stomatal density in susceptible and resistant host systems

Four host-virus systems were investigated for developmental changes in stomata density and index following viral infection. These included ‘susceptible hosts’, *Nicotiana tabacum* infected with TMV (*Tobacco mosaic virus*) and *Arabidopsis thaliana* (Col-0) infected with TVCV (*Turnip vein clearing virus*). The ‘resistant hosts’, *Nicotiana glutinosa* and *Chenopodium quinoa* plants infected with TMV were also investigated. Both viruses exhibit systemic infections in their respective susceptible hosts, and symptoms appear 2–3 weeks after inoculation. Infected *N. tabacum* plants displayed mosaic symptoms in leaves developed after infection (systemic leaves) while after the same period, infected *A. thaliana* plants displaying leaf stunting in a systemic manner. Infections in resistant hosts are not systemic but localised, as virus-infected cells that are confined to small areas known as ‘local lesions’, which prevent the virus from spreading to the rest of the plant.

Duplicate sets of plants were inoculated with either the virus or sterile water (mock inoculation). After 7–21 days, a leaf which developed after infection, ‘systemic leaf’, was selected from each individual, typically the 2^nd^ or 3^rd^ leaf to develop since inoculation; leaf impressions were taken of the abaxial surface and images were acquired from 3 areas of each leaf and SD (stomatal density = total number of stomata per mm^2^) and SI (stomatal index = {(no. of stomata)/(no. of epidermal cells + no. of stomata)}*100) were calculated.

**Figure 1:**
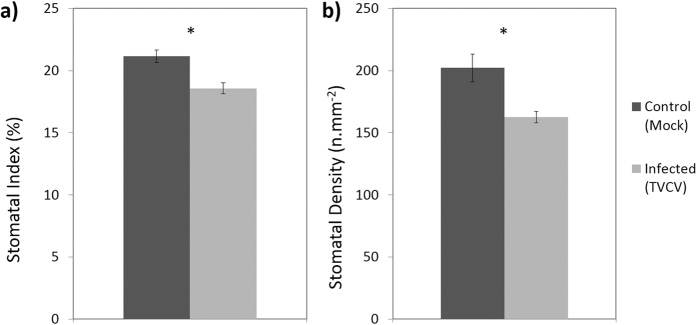
(**a**) Stomatal index and (**b**) density of systemic leaves (2^nd^ or 3^rd^ leaf developed leaf since inoculation) in *A. thaliana* WT (Col-0) plants 21 dpi with TVCV or sterile water (mock inoculation). Data presented are pooled from 3 biological replicates, significant differences (P < 0.05) are denoted by ‘*’. Bars show standard error.

**Figure 2:**
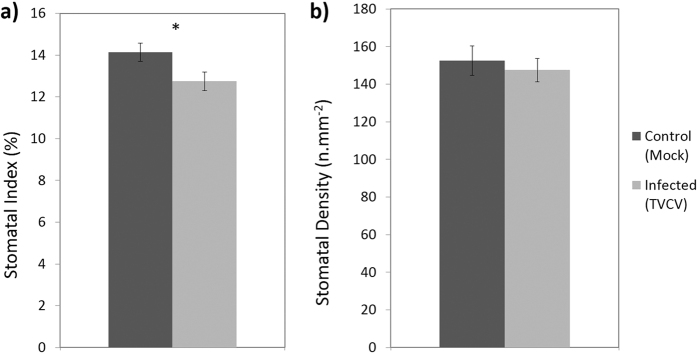
(**a**) Stomatal index and (**b**) density of systemic leaves (2^nd^ or 3^rd^ leaf developed leaf since inoculation) in *N. tabacum* plants 21 dpi with TMV or sterile water (mock inoculation). Data presented are pooled from 3 biological replicates, significant differences (P < 0.05) are denoted by ‘*’ and bars show standard error.

Susceptible hosts displayed a reduction in SI (both host-virus systems; Figs 1a and 2a) and in *A. thaliana* a reduction in SD was also observed (Fig. 1b). In *A. thaliana* TVCV-infected plants SI was reduced by 19.6% (*n* = 42–45, P < 0.001; Fig. 1a), and SD was reduced by 12.3% (*n* = 42–45, P = 0.001; Fig. 1b) when compared with mock inoculated control plants 21 dpi (days post inoculation). Likewise, a 9.8% reduction in SI was also observed in TMV-infected *N. tabacum (n* = 48, P = 0.026; Fig. 2a), and whilst a very small reduction was also observed in SD this was not significant (*n* = 48, P = 0.614; Fig. 2b).

**Figure 3:**
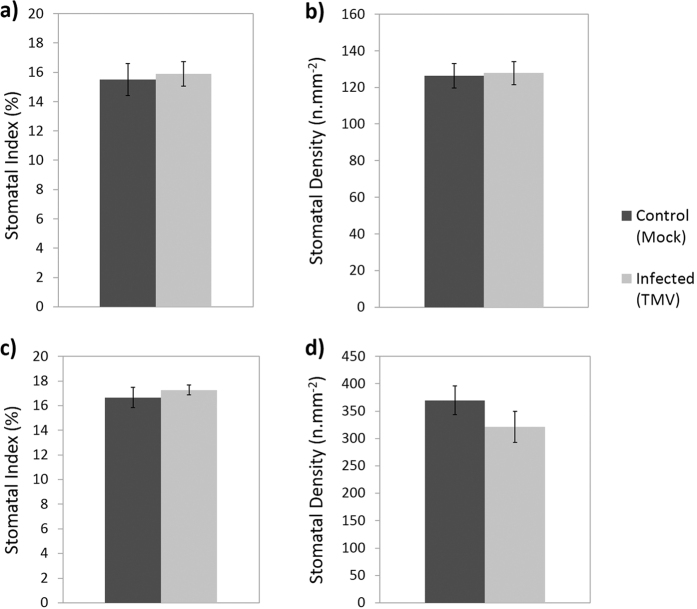
(**a**,**b**) Stomatal index (**a**) and density (**b**) of systemic leaves (2^nd^ or 3^rd^ leaf developed leaf since inoculation) from *N. glutinosa* plants 7 dpi with TMV or sterile water (mock inoculation). (**c**,**d**) Stomatal index (**c**) and density (**d**) of systemic leaves (2^nd^ or 3^rd^ leaf developed leaf since inoculation) from *C. quinoa* plants 7 dpi with TMV or sterile water (mock inoculation). Data presented are from a single biological repeat per species, bars show standard error.

In contrast to the susceptible hosts, the resistant hosts *N. glutinosa* and *C. quinoa* did not exhibit a reduction in SI (*N. glutinosa: n* = 18, P = 0.581; Fig. 3a, *C. quinoa*: n = 17–19, P = 0.457; Fig. 3c) or SD (*N. glutinosa: n* = 18, P = 0.872; Fig. 3b, *C. quinoa: n* = 17–19, P = 0.215; Fig. 3d) in the systemic leaves when the plants were infected with TMV 7 dpi. This revealed that in marked contrast to the susceptible host there was no significant change in either SD or SI in the systemic leaves, which remain virus free (Fig. 3a–d).

### Stomatal length is unaffected by viral infection

**Figure 4:**
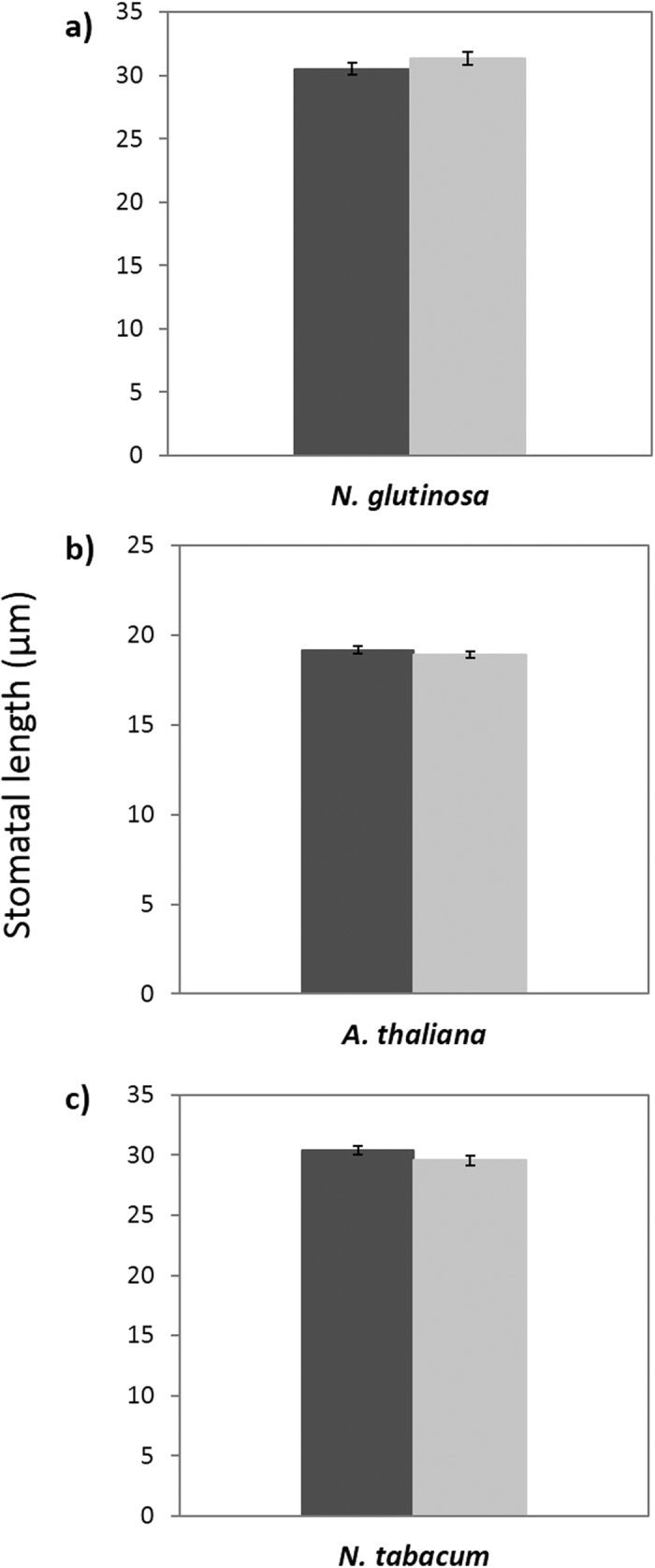
Mean size of stomata (length) in systemic (2^nd^ or 3^rd^ leaf developed leaf since inoculation) leaves of healthy (mock inoculated) and infected (TMV or TVCV) *N. glutinosa* (7 dpi) (**a**), *A. thaliana* (21 dpi) (**b**) and *N. tabacum* (21 dpi) (**c**) plants. Data presented are from a single data set per species, bars show standard error.

Stomatal length was also analysed in this investigation. There was no difference in stomatal length infected or uninfected *N. glutinosa* (resistant to systemic infection) (*n* = 14, P = 0.214; Fig. 4a). Neither was there a difference in length in systemic leaves of susceptible hosts, *A. thaliana* and *N. tabacum (A*. thaliana: *n* = 15, P = 0.397: Fig. 4b, *N. tabacum: n* = 20, P = 0.116; Fig. 4c).

### Transpiration assays

**Figure 5:**
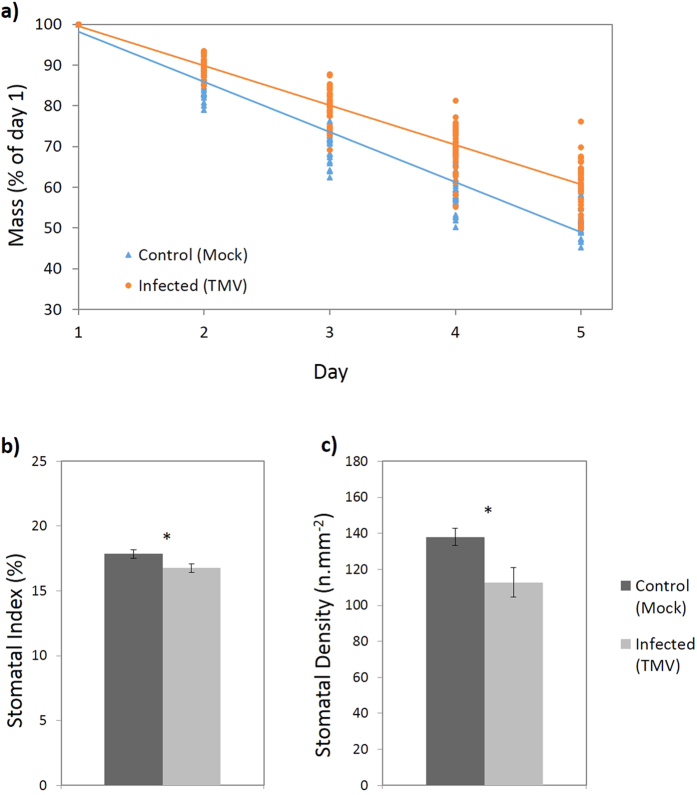
(**a**) Mass of individual plants with pots sealed around the stem (∴water loss is equivalent to transpiration) over the course of 5 days. *N. tabacum* plants were either inoculated with TMV or sterile water (mock inoculation) and measurements began 14 dpi. Data presented are pooled from 2 biological replicates, points are individual plant recordings. (**b**,**c**) Stomatal index (**b**) and density (**c**) of systemic leaves (2^nd^ or 3^rd^ leaf developed leaf since inoculation) from the plants measured in this experiment. Bars show standard error and ‘*’ denotes a significant difference (P < 0.05).

In order to investigate whether viral-induced changes in stomatal development impact on plant water relations transpiration rates were measured in *N. tabacum* healthy and TMV-infected plants in a separate experiment to those described above. TMV-infected *N. tabacum* plants exhibited significantly lower rate of water loss (*n* = 35, P < 0.001; Fig. 5a) consistent with a lower rate of transpiration, measured at 14 dpi. A subset of these plants was also measured for SI/SD values which were lower in TMV-infected plants for both SI (*n* = 20, P = 0.0004; Fig. 5b) and SD (*n* = 20, P = 0.0243; Fig 5c).

## Discussion

Stomatal development is influenced by a range of environmental cues and local signals^4^,^5^,^16^. Recently there has been considerable attention paid to the role of stomata during infection particularly by fungi and bacteria^15^. However much less attention has been devoted to the effects of viral infection on stomatal development. Previous investigations have briefly noted some viral induced developmental changes, in that *Sweet potato feathery mottle virus* (SPFMV) infected sweet potato plants have fewer, smaller, stomata^17^, while strawberry plants infected with a trio of viruses (*Strawberry crinkle virus* (SCrV), *Strawberry mottle virus* (SMV) and *Strawberry mild yellow edge virus* (SMYEV)) had fewer stomata^18^. In addition tobacco plants infected with a novel TMV strain from Egypt had fewer stomata^19^ and finally Hall & Loomis (1972) reported a reduction in stomatal numbers on the upper surfaces of *Beta vulgaris* L. leaves when infected with *Beet yellow virus* (BYV)^20^.

Ruggenthaler *et al*.^21^ first demonstrated a possible molecular link between plant virus infections and stomatal development^21^. When the gene for the protein AtMBP2C (host protein shown to associate with the movement protein of TMV) was overexpressed in *A. thaliana* the mutants showed abnormal stomatal patterning. This was apparent with increased levels of paired stomata compared with WT, and low levels of triplet and quartette stomatal clusters were also found in the mutant^21^.

Here we extend these studies and present insights into the physiological responses during viral infection, likely as a consequence of altered stomatal development. We show that viral infection in two susceptible host types; *A. thaliana* and *N. tabacum* infected with TVCV and TMV respectively is associated with a reduction in stomatal density (*Arabidopsis*) and index (both systems) and that this is a systemic response. In marked contrast in the resistant hosts, *N. glutinosa* and *C. quinoa* infected with TMV, there was no significant change in stomatal density or index in virus free systemic leaves of mock and virus inoculated plants. We also showed that a reduction in transpiration was observed in virus infected *N. tabacum* that exhibited reduced stomatal index/density at 2 weeks post inoculation.

The question of whether the reduction in stomatal index seen in the infection on susceptible hosts is part of a defence response by the plant or is of benefit to the virus during infection will be the subject of future investigations. In the absence of other compensatory factors a reduction in stomatal density and index would likely be associated with reduced transpiration and possible increased host water use efficiency^10^,^11^,^12^,^22^.

A reduction in transpiration could also lead to reduced viral movement around the host. Viruses such as TMV are known to travel long distances through the phloem^23^. However, increasingly plant viruses are being found in the xylem and are capable of generating systemic infection via xylem transport^24^,^25^. In addition, transpiration rate will affect the movement of the virus through the apoplastic pathway and horizontal transportation from the phloem into the surrounding tissues^26^. A reduction in stomatal index could also be a response from the host to prevent further infection. Many pathogens use stomata as an entry portal to the plant host including bacteria, fungi, nematodes and protists^27^.

In summary, here we report that during viral infection, in susceptible hosts, there is a decrease in the number of stomata that develop on the leaf surface of leaves that have developed post-infection indicating that this is a systemic response.

## Methods

### Plant material and virus isolates

*Nicotiana* and *Chenopodium* spp were allowed to germinate on soil after which they were transplanted into individual pots and grown under glasshouse conditions using natural lighting supplemented with artificial illumination under a natural photoperiod. Temperatures in the glasshouse were between 16–23 °C. *Arabidopsis thaliana* WT (Col-0) and mutant plants were germinated on soil and were transplanted 7 days after germination. These plants were grown under short day light cycles (8:16, day:night) at 22 °C and at a light intensity of 112 μmol m^−2 ^s^−1^. All seeds were obtained from the University of Bristol Experimental Glasshouses.

The TMV (*Tobacco mosaic virus*) isolate used was TMV U1 (UK) from *N. tabacum* infected leaves. TVCV (*Turnip vein-clearing virus*) was kindly donated by John Carr (Department of Plant Sciences, University of Cambridge) from infected *N. tabacum* material.

### Virus inoculation methods

Virus inoculum was prepared for manual inoculations by homogenising a small amount of infected leaf tissue (*N. tabacum* leaves for TMV, *N. benthamiana* leaves for TVCV) in sterile water. When plants were either 3–4 weeks old (*A. thaliana*) or 4–6 weeks old (*Nicotiana* spp), a healthy leaf was then chosen for inoculation, typically 2^nd^ or 3^rd^ newest leaf, which was then labelled by piercing a small hole using a sterile pipette tip, and dusted with carborundum powder. The virus homogenate was applied by gently stroking it onto the leaf with a gloved finger. After several minutes the inoculated leaf was washed with sterile water and the plant was left at specified growth conditions to allow development of infection. Mock inoculations received the same treatment but sterile water was used instead of virus inoculum.

### Stomatal development analysis

Dental resin (President Jet Light Body, Coltène/Whaledent, Burgess Hill, UK) was applied to the underside of the 3^rd^ or 4^th^ leaf that had developed since inoculation. In susceptible host species (*N. tabacum* and *A. thaliana*) this was 21 dpi and in resistant host species (*N. glutinosa* and *C. quinoa*) this was 7 dpi; this discrepancy was due to the nature of infections established in the respective hosts. In susceptible species, as the virus causes systemic infection and symptoms take 2–3 weeks to appear post inoculation, whereas the resistant host species produce symptoms after 1–3 dpi, as the asymptomatic leaves are virus free. Once set, the leaf was removed and colourless nail polish (Revlon) was coated on the impression. After drying, colourless sticky tape (Sellotape) was used to transfer the secondary impression onto a microscope slide and stuck down.

Slides were imaged using an inverted Zeiss Axiovert 200 M microscope driven by Volocity software (Improvision Ltd, Coventry, UK). For each slide, 3 areas were captured (top, middle and bottom). From these images, cell counts and size measurements could be made using Volocity Demo, and stomatal index (SI = {(no. of stomata)/(no. of epidermal cells + no. of stomata)}*100) and density (SD = total number of stomata per mm^2^) could be calculated. Student’s t-tests were used to asses significant differences (SI data required transformation and was subjected to arcsine square root transformation, formula used: = ASIN[SQRT(Stomatal Index/100)]) and data was tested for normality before applying the statistical test. Statistics were performed in Microsoft Excel (2010) and IBM SPSS Statistics 21.

For *A. thaliana* and *N. tabacum* plants, data was collated from 3 independent replicates into a mega-analysis. Resistant hosts *N. glutinosa* and *C. quinoa* contained compiled data from 2 biological replicates.

Stomatal size analysis was performed by measuring 15 stomata per individual plant (5 stomata per image, 3 areas captured per leaf) and the length of each stomata was recorded. Data in this section is from one biological replicate for each species. The data was tested for normality and a student’s t-test was used to assess differences in means.

### Transpiration assay

Transpiration assays were performed on *N. tabacum* plants that were mock inoculated or infected with TMV, 14 dpi. Duplicate sets of plants were either inoculated with TMV or mock inoculated with sterile water, after 14 days the pots were wrapped in plastic and sealed around the stem. Mass was recorded daily on a balance for 5 consecutive days. Measurements were recorded for each individual and regression plots were formed and homogeneity of slopes was measured using STATGAPHICS-Centurion. Data was compiled from 2 biological replicates.

## Additional Information

**How to cite this article**: Murray, R. R. *et al*. Plant virus infections control stomatal development. *Sci. Rep.* **6**, 34507; doi: 10.1038/srep34507 (2016).

## References

[b1] Hetherington A. & Woodward F. The role of stomata in sensing and driving environmental change. Nature 424, 901–908 (2003). 12931178 10.1038/nature01843

[b2] Kim T.-H. H. . Guard cell signal transduction network: advances in understanding abscisic acid, CO_2_, and Ca^2+^ signaling. Annu. Rev. Plant Biol. 61, 561–591 (2010). 20192751 10.1146/annurev-arplant-042809-112226 PMC3056615

[b3] Kollist H., Nuhkat M. & Roelfsema M. R. G. Closing gaps: linking elements that control stomatal movement. New Phytol. 203, 44–62 (2014). 24800691 10.1111/nph.12832

[b4] Casson S. A. & Hetherington A. M. Environmental regulation of stomatal development. Curr. Opin. Plant Biol. 13, 90–95 (2010). 19781980 10.1016/j.pbi.2009.08.005

[b5] Pillitteri L. J. & Torii K. U. Mechanisms of stomatal development. Annu. Rev. Plant Biol. 63, 591–614 (2012). 22404473 10.1146/annurev-arplant-042811-105451

[b6] Dow G. J. & Bergmann D. C. Patterning and processes: how stomatal development defines physiological potential. Curr. Opin. Plant Biol. 21, 67–74 (2014). 25058395 10.1016/j.pbi.2014.06.007

[b7] Lake J. A., Quick W. P., Beerling D. J. & Woodward F. I. Signals from mature to new leaves. Nature 411, 154 (2001). 11346781 10.1038/35075660

[b8] Casson S. A. . phytochrome B and PIF4 regulate stomatal development in response to light quantity. Curr. Biol. 19, 229–234 (2009). 19185498 10.1016/j.cub.2008.12.046

[b9] Casson S. A. & Hetherington A. M. phytochrome B is required for light-mediated systemic control of stomatal development. Curr. Biol. 24, 1216–1221 (2014). 24835461 10.1016/j.cub.2014.03.074 PMC4046225

[b10] Yoo C. Y. . The *Arabidopsis* GTL1 transcription factor regulates water use efficiency and drought tolerance by modulating stomatal density via transrepression of SDD1. Plant Cell 22, 4128–4141 (2010). 21169508 10.1105/tpc.110.078691 PMC3027182

[b11] Doheny-Adams T., Hunt L., Franks P. J., Beerling D. J. & Gray J. E. Genetic manipulation of stomatal density influences stomatal size, plant growth and tolerance to restricted water supply across a growth carbon dioxide gradient. Philos. Trans. R. Soc. Lond. B. Biol. Sci. 367, 547–555 (2012). 22232766 10.1098/rstb.2011.0272 PMC3248714

[b12] Franks P. J., Doheny-Adams T. W., Britton-Harper Z. J. & Gray J. E. Increasing water-use efficiency directly through genetic manipulation of stomatal density. New Phytol. 207, 188–195 (2015). 25754246 10.1111/nph.13347

[b13] Melotto M., Underwood W., Koczan J., Nomura K. & He S. Y. Plant stomata function in innate immunity against bacterial invasion. Cell 126, 969–980 (2006). 16959575 10.1016/j.cell.2006.06.054

[b14] Zeng W. Q., Melotto M. & He S. Y. Plant stomata: a checkpoint of host immunity and pathogen virulence. Curr. Opin. Biotechnol. 21, 599–603 (2010). 20573499 10.1016/j.copbio.2010.05.006 PMC2946497

[b15] McLachlan D., Kopischke M. & Robatzek S. Gate control: guard cell regulation by microbial stress. New Phytol. 203, 1049–1063 (2014). 25040778 10.1111/nph.12916

[b16] Bergmann D. C. & Sack F. D. Stomatal development. Annu. Rev. Plant Biol. 58, 163–181 (2007). 17201685 10.1146/annurev.arplant.58.032806.104023

[b17] El-Deeb S. H. & IsHak J. Histological and cytological changes due to the infection with Sweetpotato feathery mottle virus (SPFMV). Zeitschrift Fur Pflanzenkrankheiten Und Pflanzenschutz-Journal Plant Dis. Prot. 111, 247–256 (2004).

[b18] Angelov B., Kovachevan & Tsonev T. Physiological and cytological characteristics of virus affected plants of *Fragaria*-sp. Fiziol. na Rast. 16, 50–55 (1990).

[b19] Sayed E. T., Soweiha H. E. & El-Shamy M. M. Anatomical and cytopathological alterations induced by a strain of Tobacco mosaic virus in tobacco leaf tissues. Egypt. J. Microbiol. 36, 329–348 (2001).

[b20] Hall A. E. & Loomis R. S. An explanation for the difference in photosynthetic capabilities of healthy and Beet yellows virus-infected sugar beets (*Beta vulgaris* L.). Plant Physiol. 50, 576–580 (1972). 16658220 10.1104/pp.50.5.576 PMC366193

[b21] Ruggenthaler P., Fichtenbauer D., Krasensky J., Jonak C. & Waigmann E. Microtubule-associated protein AtMPB2C plays a role in organization of cortical microtubules, stomata patterning, and Tobamovirus infectivity. Plant Physiol. 149, 1354–1365 (2009). 19074626 10.1104/pp.108.130450 PMC2649411

[b22] Yoo C. Y., Pence H. E., Hasegawa P. M. & Mickelbart M. V. Regulation of transpiration to improve crop water use. CRC. Crit. Rev. Plant Sci. 28, 410–431 (2009).

[b23] Seron K. & Haenni A. L. Vascular movement of plant viruses. Mol. Plant-Microbe Interact. 9, 435–442 (1996). 8755620 10.1094/mpmi-9-0435

[b24] Otulak K. & Garbaczewska G. Cytopathological Potato virus Y structures during *Solanaceous* plants infection. Micron 43, 839–850 (2012). 22410276 10.1016/j.micron.2012.02.015

[b25] Wan J., Cabanillas D. G., Zheng H. & Laliberté J.-F. Turnip mosaic virus moves systemically through both phloem and xylem as membrane-associated complexes. Plant Physiol. 167, 1374–1388 (2015). 25717035 10.1104/pp.15.00097 PMC4378181

[b26] De Schepper V., De Swaef T., Bauweraerts I. & Steppe K. Phloem transport: a review of mechanisms and controls. J. Exp. Bot. 64, 4839–4850 (2013). 24106290 10.1093/jxb/ert302

[b27] Grimmer M. K., Foulkes M. J. & Paveley N. D. Foliar pathogenesis and plant water relations: a review. J. Exp. Bot. 63, 4321–4331 (2012). 22664583 10.1093/jxb/ers143

